# Early genetic testing in pediatric epilepsy: Diagnostic and cost implications

**DOI:** 10.1002/epi4.12878

**Published:** 2023-12-16

**Authors:** Shanna M. Swartwood, Ana Morales, Kathryn E. Hatchell, Chad Moretz, Dianalee McKnight, Laurie Demmer, Sarah Chagnon, Swaroop Aradhya, Edward D. Esplin, Joshua L. Bonkowsky

**Affiliations:** ^1^ Division of Pediatric Neurology, Department of Pediatrics University of Utah School of Medicine Salt Lake City Utah USA; ^2^ Invitae Corporation San Francisco California USA; ^3^ Division of Medical Genetics, Department of Pediatrics, Atrium Health's Levine Children's Hospital Charlotte North Carolina USA; ^4^ Division of Child and Adolescent Neurology, Children's Hospital of the Kings Daughters Virginia; ^5^ Center for Personalized Medicine, Primary Children's Hospital Salt Lake City Utah USA

**Keywords:** epilepsy, genetic, healthcare costs, multigene epilepsy panel, pediatric

## Abstract

The identification of numerous genetically based epilepsies has resulted in the widespread use of genetic testing to inform epilepsy etiology. Our study aims to investigate whether a difference exists in the diagnostic evaluation and healthcare‐related cost expenditures of pediatric patients with epilepsy of unknown etiology who receive a genetic diagnosis through multigene epilepsy panel (MEP) testing and comparing those who underwent early (EGT) versus late genetic testing (LGT). Testing was defined as early (less than 1 year), or late (more than 1 year), following clinical epilepsy diagnosis. A retrospective chart review of pediatric individuals (1–17 years) with epilepsy of unknown etiology who underwent multigene epilepsy panel (MEP) testing identified 28 of 226 (12%) individuals with a pathogenic epilepsy variant [EGT *n* = 8 (29%); LGT *n* = 20 (71%)]. The average time from clinical epilepsy diagnosis to genetic diagnosis was 0.25 years (EGT), compared with 7.1 years (LGT). The EGT cohort underwent fewer metabolic tests [EGT *n* = 0 (0%); LGT *n* = 16 (80%) (*P* < 0.01)] and invasive procedures [EGT *n* = 0 (0%); LGT *n* = 5 (25%) (*P* = 0.06)]. Clinical management changes implemented due to genetic diagnosis occurred in 10 (36%) patients [EGT *n* = 2 (25%); LGT *n* = 8 (40%) (*P* = 0.76)]. Early genetic testing with a MEP in pediatric patients with epilepsy of unknown etiology who receive a genetic diagnosis is associated with fewer non‐diagnostic tests and invasive procedures and reduced estimated overall healthcare‐related costs.

**Plain language summary:**

This study aims to investigate whether a difference exists in the diagnostic evaluation and cost expenditures of pediatric patients (1‐17 years) with epilepsy of unknown cause who are ultimately diagnosed with a genetic cause of epilepsy through multigene epilepsy panel testing and comparing those who underwent early testing (less than 1 year) versus late testing (more than 1 year) after clinical epilepsy diagnosis. Of the 28 of 226 individuals with a confirmed genetic cause of epilepsy on multigene epilepsy panel testing, performing early testing was associated with fewer non‐diagnostic tests, fewer invasive procedures and reduced estimated overall healthcare‐related costs.

**Key Points**

- The clinical utility and cost mitigation associated with early genetic testing for pediatric patients with epilepsy of unknown etiology is an unexplored question.
- Early genetic testing (within 1 year of clinical epilepsy diagnosis) with a multigene epilepsy panel in pediatric patients who receive a genetic diagnosis is associated with fewer non‐diagnostic tests and invasive procedures.
- Early identification of genetically based epilepsies may reduce overall healthcare‐related costs and augment initiation of targeted treatment strategies.

## 1 INTRODUCTION

Epilepsy is one of the most common chronic neurological conditions seen in pediatric patients affecting roughly 0.5%–1% of the population.^1^ The identification of epilepsy etiology is a critical component of epilepsy management as it aids in prognostic counseling, surveillance of comorbidities and the potential for the implementation of targeted treatment strategies.^2^ The advent of advanced diagnostic tools, particularly in the field of genetics, has resulted in the discovery of numerous genetically based epilepsies.^3^ Compared with the previous era of single‐gene testing in epilepsy, which yielded a diagnosis in <5% of cases, next‐generation sequencing typically results in a genetic diagnosis in 10% or more of patients tested with a diagnostic yield closer to 30% in those with an underlying epileptic encephalopathy.^4^ The increasing identification of genetic epilepsies has shifted treatment practices toward precision‐based medicine strategies.^3^

Despite the potential for rapid genetic screening tests for epilepsy, including targeted multigene epilepsy panels (MEP), an extensive and often step‐wise diagnostic evaluation is regularly undertaken at the onset of epilepsy diagnosis, which can include invasive procedures and incur substantial healthcare costs.^5^ The clinical utility and healthcare‐related cost mitigation associated with implementing an early genetic testing strategy for pediatric patients with epilepsy of unknown etiology is an unexplored question. Our study aims were to investigate whether a difference exists in the diagnostic evaluation and healthcare‐related cost expenditures of pediatric patients with epilepsy of unknown etiology who receive a genetic diagnosis through early versus late genetic testing with a multigene epilepsy panel.

## 2 METHODS

This study was a retrospective chart review of pediatric patients (1–17 years) diagnosed with epilepsy at Primary Children's Hospital in Salt Lake City, Utah, Atrium Health Levine Children's in Charlotte, North Carolina, and Children's Hospital of the King's Daughters in Norfolk, Virginia, from October 2016 to July 2019. Ethical approval was obtained for this work (WCG IRB protocol #1167406). Eligible cases were identified based on the following criteria: clinically confirmed epilepsy defined as having at least two unprovoked seizures occurring more than 24 h apart or one unprovoked seizure with a propensity for others,^6^ meeting age requirements, unknown etiology for epilepsy, actively followed for at least 1 year after epilepsy diagnosis with medical records documented in the electronic medical record (EMR) since presentation and received genetic testing with a MEP (Invitae® Epilepsy Panel, at least 133 genes associated with syndromic and non‐syndromic causes of epilepsy). Of the identified eligible patients, only those with a definitive molecular diagnosis identified from MEP testing suspected to be the etiology for epilepsy were included in the final cohort. A definitive molecular diagnosis was defined as either a single pathogenic variant (P) or likely pathogenic variant (LP) in a gene associated with autosomal dominant (AD), X‐linked, or two P/LP variants (or a single homozygous variant) in genes associated with autosomal recessive (AR) inheritance.^7^ Exclusion criteria consisted of seizures not meeting criteria for epilepsy, established etiological diagnosis for epilepsy prior to MEP testing, genetic testing with MEP not performed and/or not diagnostic including negative result, benign variant, likely benign variant, a single P/LP variant in genes associated with AR inheritance (carrier), phenotype suggestive of a triplet repeat expansion, genetic deoxyribonucleic acid (DNA) samples from fetal cells or non‐blood sources and genetic samples with known mosaicism.

Patients were categorized into two distinct cohorts, those who underwent early genetic testing with a MEP within 1 year of clinical epilepsy diagnosis (EGT) and those who underwent testing beyond 1 year (LGT). Electronic case report forms of eligible patients were completed to capture the following characteristics: age at seizure onset, drug‐resistant epilepsy (DRE) defined as having unprovoked breakthrough seizures despite treatment with two or more appropriately prescribed and dosed antiseizure medications (ASM),^8^ type and number of genetic tests performed, serum, and/or urine diagnostic testing for metabolic disorders, lumbar puncture, unscheduled hospitalizations (average number from 12‐month period prior to chart review excluding planned electroencephalogram [EEG] monitoring admissions), emergency department (ED) visits for epilepsy‐related reasons and changes in epilepsy management based on MEP testing results which included the following categories: started, changed, added, or stopped ASM therapy, dietary modification, surgery, and/or neurostimulation device recommended, monitoring for extra‐neurological disease and referral to a specialist. Due to limited available data regarding healthcare costs in the study sample, published literature was used to estimate healthcare‐related costs and potential cost‐savings.^6^, ^9^ To minimize geographic and insurance coverage induced biases, standardized costs per test and diagnostic procedure were assigned across all patients consistent with the methodologic approach used in prior analogous studies.^10^, ^11^

### 2.1 Statistical analyses

Descriptive statistics were used to characterize the study cohort with data presented as frequency and percent, unless otherwise specified. The prop. test (non‐parametric) in R studio (2022.07.0) was utilized for assessing differences in the proportions between the EGT versus LGT cohorts for the following comparisons: prior genetic testing, clinical management changes, metabolic testing, and invasive procedures. The Mann–Whitney *U* test (wilcox. test) was used to compare number of ED visits and number of unscheduled hospitalizations for epilepsy‐related concerns between groups. A *P‐*value < 0.05 was assumed to indicate a statistically significant difference.

## 3 RESULTS

**FIGURE 1:**
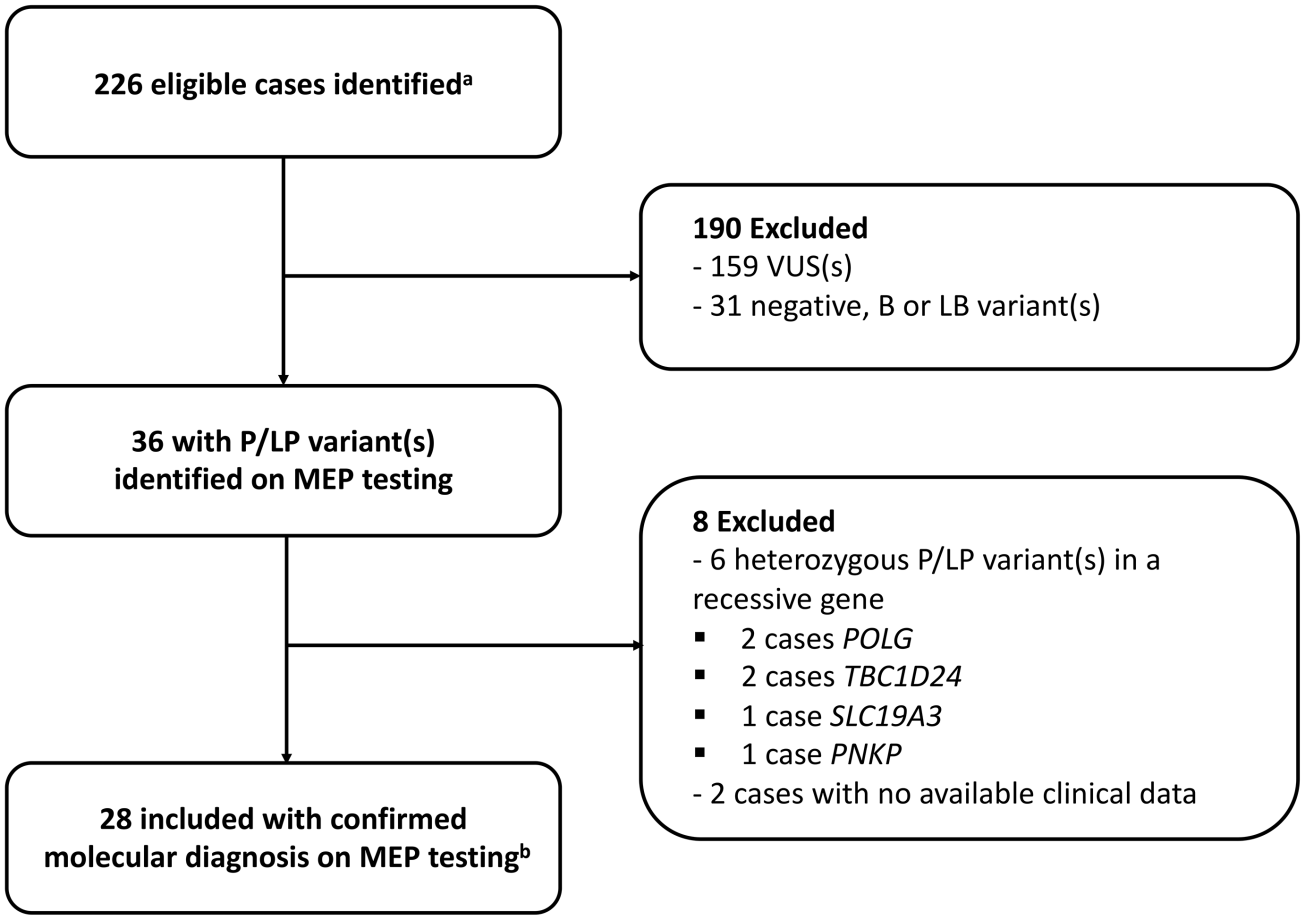
Patient selection. B, benign; LB, likely benign; LP, likely pathogenic; MEP, multigene epilepsy panel; P, pathogenic; VUS, variant(s) of uncertain significance. (a) Eligible cases included patients meeting the following criteria: pediatric patient (1–17 years), clinically confirmed epilepsy, unknown etiology for epilepsy prior to MEP testing, received genetic testing with a MEP and actively followed for at least 1 year after epilepsy diagnosis with medical records documented in the electronic medical record since presentation. (b) Molecular diagnosis was defined as either a single pathogenic variant (P) or likely pathogenic variant (LP) in a gene associated with autosomal dominant (AD), X‐linked, or two P/LP variants (or a single homozygous variant) in genes associated with autosomal recessive (AR) inheritance.

The identification of the study population is outlined in Figure 1. From a total of 226 eligible cases, after accounting for inclusion and exclusion criteria, a total of 28 (12%) cases comprised the final cohort with a confirmed genetic cause of epilepsy based on MEP testing results [EGT = 8 (29%); LGT = 20 (71%)]. The specific genetic variants identified in the final cohort is available in Table S1. Demographics and clinical characteristics are summarized in Table 1. The average age at seizure onset for the entire cohort was 1.51 years (EGT = 1.25 years; LGT = 1.78 years); median age of seizure onset was 1 year for the entire cohort and each subgroup. Most patients had DRE (19 [68%]) with similar percentages in the EGT (*n* = 5 [63%]) and LGT (*n* = 14 [70%]) cohorts. The average time from clinical diagnosis to identification of genetic etiology of epilepsy was less for the EGT cohort (0.25 years, median 0.5, range 0–1) compared with the LGT cohort (7.1 years, median 5.0, range 2–17). Of the entire cohort, the initial genetic test performed was a MEP in 16 (57%) patients [EGT = 7 (88%); LGT = 9 (45%)]. The remaining 12 (43%) patients [EGT = 1 (13%); LGT = 11 (92%)] underwent an alternative non‐diagnostic genetic test prior to MEP including CMA (6 [50%]), karyotype (3 [25%]), single‐gene panel (1 [8%]), alternative non‐epilepsy specific multigene panel (1 [8%]) and other (1 [8%]).

**TABLE 1:** Demographic, diagnostic and epilepsy characteristics of patient cohort.

Characteristic	Total (*n* = 28)	EGT (*n* = 8)	LGT (*n* = 20)
Gender, *n* (%)			
Male	11 (39)	5 (63)	12 (60)
Female	17 (61)	3 (37)	8 (40)
Race, *n* (%)			
White	17 (61)	5 (63)	12 (60)
Hispanic	2 (7)	1 (12)	1 (5)
Asian	2 (7)	1 (12)	1 (5)
Black/African American	4 (14)	1 (12)	3 (15)
Unknown	3 (11)	0 (0)	3 (15)
Age at seizure onset (y)			
Average	1.5	1.3	1.8
Median	1.0	1.0	1.0
Range	0–7	0–4	0–7
Drug‐resistant epilepsy, *n* (%)	19 (68)	5 (63)	14 (70)
Time from epilepsy diagnosis to genetic diagnosis (y)			
Average	5.0	0.25	7.1
Median	3.0	0.50	5.0
Range	0–17	0–1	2–17
Initial genetic test performed, *n* (%)			
MEP	15 (53)	7 (87)	8 (40)
CMA	6 (21)	0 (0)	6 (30)
Karyotype	3 (11)	1 (13)	2 (10)
Single‐gene panel	1 (4)	0 (0)	1 (5)
Other	3 (11)	0 (0)	3 (15)
Metabolic serum/urine testing, *n* (%)	16 (57)	0 (0)	16 (80)
Invasive procedure (LP), *n* (%)	5 (18)	0 (0)	5 (25)
Epilepsy‐related unscheduled hospitalizations			
Average	1.9	1.5	2.0
Median	1.5	1.0	1.5
Range	0–12	0–3	0–12
Epilepsy‐related ED visits			
Average	4.3	3.1	4.8
Median	3.0	3.0	3.0
Range	0–20	1–6	0–20
Clinical management changes due to MEP results, *n* (%)			
Initiation of medication	5 (18)	1 (13)	4 (20)
Discontinuation of medication	1 (4)	0 (0)	1 (5)
Avoidance of certain medication classes	1 (4)	1 (13)	0 (0)
Referral to a specialist	3 (11)	0 (0)	3 (15)
None	18 (64)	6 (75)	12 (60)

Abbreviations: CMA, chromosomal microarray; ED, emergency room; EGT, early genetic testing; LGT, late genetic testing; LP, lumbar puncture; MEP, multigene epilepsy panel.

Metabolic testing was performed as part of the diagnostic evaluation in 16 (57%) patients. A statistically significant difference was identified among the EGT versus LGT cohorts with no individuals in the EGT cohort undergoing metabolic testing versus 16 (80%) patients in the LGT cohort (*P* < 0.01). An invasive procedure, which was classified as the collection of cerebrospinal fluid (CSF) through a lumbar puncture, was performed in 5 (18%) patients in the total cohort, all of which were in the LGT cohort. No patients in the EGT cohort underwent an invasive procedure as part of the diagnostic evaluation (*P* = 0.06) (Table 1).

The average number of unscheduled hospitalizations in the 12‐month period prior to chart review for epilepsy‐related concerns, excluding planned EEG monitoring admissions was 2.1 visits for the LGT cohort (median 1.5, range 0–12) compared with 1.5 visits for the EGT cohort (median 1.0, range 0–3) (*P* = 0.77). The average number of ED visits for epilepsy‐related concerns was 4.8 visits for the LGT cohort (median 3.0, range 0–20) compared with 3.1 visits for the EGT cohort (median 3.0, range 1–6) (*P* = 0.90) (Table 1).

A change in clinical management due to MEP testing results occurred in a total of 10 (36%) patients [EGT = 2 (25%); LGT = 8 (40%) (*P* = 0.76)]. Clinical management changes included: initiation or addition of a new medication [EGT = 1 (12.5%); LGT = 4 (20%)], cessation of a medication [LGT = 1 (5%)], avoidance of certain medication classes [EGT = 1 (12.5%)], and referral to a specialist [LGT = 3 (15%)] (Table 1).

## 4 DISCUSSION

Our data suggest that early genetic testing in pediatric patients with epilepsy of unknown etiology who receive a genetic diagnosis is associated with fewer non‐diagnostic tests and invasive procedures and reduced estimated overall healthcare‐related costs. A recent study found that genetic diagnoses in patients with epilepsy was associated with changes in clinical management in 49.8% of individuals and usually (81.7% of the time) within 3 months of receiving the result.^2^ Thus early MEP testing may have implications for prompt changes to clinical care, including implementation of targeted treatment strategies.^3^

Non‐diagnostic metabolic testing and invasive procedures occurred more frequently in those individuals who underwent late compared with early genetic testing after clinical epilepsy diagnosis. Based on the reduction in other diagnostic testing, our findings suggest that early genetic testing is associated with lower healthcare costs. Using publicly available test cost data for each of the tests in this analysis, we assigned an estimated cost (i.e., metabolic tests [plasma amino acids—$350, carnitine studies—$350, urine organic acids—$947, ammonia level—$80, lactic acid—$100, very long chain fatty acids (VLCFA)—$230, creatinine kinase—$50, CSF immunoglobulins—$100, CSF neurotransmitters—$100, CSF lactate—$100, CSF amino acids—$100, methylmalonic acid—$100, homocysteine—$80, pyruvate—$168, biotinidase—$215, carbohydrate‐deficient transferrin glycoprotein—$200], genetic tests [chromosome microarray—$2100, karyotype—$1100, single‐gene test—$2300, multigene panel—$3675, HLA test—$100], lumbar puncture for CSF—$2572, brain MRI—$3500, electroencephalogram [EEG]—$950, 24 h EEG—$2823).^12^, ^13^, ^14^, ^15^ Based on these cost estimates, we compared the LGT and EGT cohorts. The estimated cost of previous non‐diagnostic genetic testing in the LGT cohort was $29 925 ($1496 per patient) compared with $3200 ($400 per patient) in the EGT cohort.^12^, ^13^ The estimated cost for metabolic testing was $24 816 ($1241 per patient)^14^ and lumbar puncture was $10 288 ($514 per patient) in the LGT cohort.^15^ No metabolic testing or lumbar puncture was indicated or performed in the EGT cohort. Thus, the cost of non‐diagnostic testing was $3251 per patient ($65 029 total) in the LGT cohort, compared with $400 per patient ($3200 total) for the EGT cohort suggesting that upfront genetic testing with MEP at time of epilepsy diagnosis may result in a potential savings of $2208 per patient ($61 829 total).

Universal genetic testing for pediatric patients with unknown epilepsy etiology, as recommended recently by the National Society of Genetic Counselors (NSGC) practice guidelines,^16^ should be considered early after clinical epilepsy diagnosis. Given recent advances in next‐generation sequencing technologies and reductions in costs of testing,^17^ universal genetic testing for the pediatric epilepsy population, particularly patients with DRE, is a reasonable goal of management.

### 4.1 Limitations and future directions

Limitations of this study are its retrospective nature, small sample size and lack of analysis of patients with non‐diagnostic MEP testing results. The diagnostic yield of MEP testing in our cohort was 12%, which is slightly lower than the previously published positivity rate for MEP testing.^4^, ^18^ Multiple studies report an increased diagnostic yield when MEP testing is performed in those less than 1 year of age and those with an epileptic encephalopathy.^18^ The small sample size of our cohort may under‐represent these specific patient populations leading to our lower diagnostic yield. Finally, the authors recognize that performing early genetic testing will inherently alter the course of subsequent diagnostic evaluation; however, the retrospective nature of this study limits further comparisons. In addition, only patients with a positive diagnostic result from MEP testing were included, thus conclusions can only be drawn regarding these individuals. A prospective study design with a larger patient cohort investigating early versus late genetic testing in pediatric patients with epilepsy of unknown etiology and comparing cost and clinical outcomes of individuals with diagnostic and non‐diagnostic genetic testing may prove useful.

## 5 CONCLUSIONS

Early genetic testing with a MEP in pediatric patients with epilepsy of unknown etiology who receive a genetic diagnosis is associated with decreased non‐diagnostic tests, fewer invasive procedures and reduced estimated healthcare‐related costs. The earlier identification of genetically based epilepsies may augment initiation of targeted treatment strategies.

## AUTHOR CONTRIBUTIONS

All co‐authors have been substantively involved in the study and/or preparation of the manuscript. All authors participated in drafting the manuscript and in final approval of the manuscript for publication.

## CONFLICT OF INTEREST STATEMENT

The following authors are employees and shareholders at Invitae Corporation: Esplin, Morales, Aradhya, Hatchell, Moretz, and McKnight. The remaining authors have no conflicts of interest to disclose.

## ETHICAL APPROVAL

We confirm that we have read the Journal's position on issues involved in ethical publication and affirm that this report is consistent with those guidelines.

## Supporting information


Table S1.

## Data Availability

The data that support the findings of this study are available upon request from the corresponding author. The data are not publicly available due to privacy or ethical restrictions.
