# Supplementary material for: Early genetic testing in pediatric epilepsy: Diagnostic and cost implications
Source: Epilepsia Open. 2023 Dec 16;9(1):439–44. doi: 10.1002/epi4.12878 (PMC10839360; doi:10.1002/epi4.12878)
Supplement: Supplementary file 1 — Table S1. [file EPI4-9-439-s001.docx]

**Supplementary Table 1: Pathogenic and Likely Pathogenic Variants Identified in Patient Cohort**

| **Gene** | **Genetic Variant** | **Effect** | **Interpretation** |
| --- | --- | --- | --- |
| *ALG13* | NM_001257230.1:c.8A>G | p.Asn3Ser | Pathogenic |
| *ARX* | NM_139058.2:c.426_458dup | p.Gly143_Ala153dup | Pathogenic |
| *CACNA1A* | NM_001127221.1:c.4953+1G>T | Splice donor | Pathogenic |
| *CHD2* | NM_001271.3:c.2765dup | p.Glu923Glyfs*42 | Pathogenic |
| *CLN3* | Deletion | Entire sequence | Pathogenic |
| *CLN3* | Deletion | Exons 8-9 | Pathogenic |
| *DEPDC5* | NM_001242896.1:c.1555C>T | P.Gln519* | Pathogenic |
| *DEPDC5* | NM_001242896.1:c.3563+1G>C | Splice donor | Likely Pathogenic |
| *DEPDC5* | NM_001242896.1:c.871+1G>T | Splice donor | Likely Pathogenic |
| *DEPDC5* | NM_001242896.1:c.1663C>T | p.Arg555* | Pathogenic |
| *DEPDC5* | NM_001242896.1:c.59-?_146+?del | Deletion (Exon 3) | Pathogenic |
| *DYRK1A* | NM_001396.3:c.951+4_951+7del | Intronic | Pathogenic |
| *GABRB3* | NM_000814.5:c.-114-?_*4247+?del | Deletion (Entire sequence) | Pathogenic |
| *MBD5* | NM_018328.4:c.2633delC | p.Pro878Hisfs*37 | Pathogenic |
| *NPRL3* | NM_001077350.2:c.-67-?_*904+?del | Deletion (Entire coding sequence) | Pathogenic |
| *PCDH19* | NM_001184880.1:c.1091dupC | p.Tyr366Leufs*10 | Pathogenic |
| *PCDH19* | NM_001184880.1:c.1019A>G | p.Asn340Ser | Pathogenic |
| *PNKP^* | NM_007254.3: c.1253_1269dup | p.Thr424Glyfs*49 | Pathogenic |
| *POLG^* | NM_002693.2:c.2209G>C | p.Gly737Arg | Pathogenic |
| *POLG^* | NM_002693.2:c.3287G>A | p.Arg1096His | Likely Pathogenic |
| *SCN1A* | NM_001165963.1:c.1834C>T | p.Arg612* | Pathogenic |
| *SCN1A* | NM_001165963.1:c.2831T>C | p.Val944Ala | Pathogenic |
| *SCN1A* | NM_001165963.1:c.4462delC | p.Gln1488Serfs*13 | Pathogenic |
| *SCN1A* | NM_001165963.1:c.5263G>T | p.Asp1755Tyr | Pathogenic |
| *SCN1A* | NM_001165963.1:c.5726C>T | p.Thr1909Ile | Pathogenic |
| *SCN2A* | NM_021007.2:c.685T>G | p.Ser229Ala | Likely Pathogenic |
| *SLC19A3^* | NM_025243.3:c.337T>C | p.Tyr113His | Pathogenic |
| *SLC6A1* | NM_003042.3:c.1377C>G | p.Ser459Arg | Pathogenic |
| *SLC6A1* | NM_003042.3:c.223G>A | p.Gly75Arg | Likely Pathogenic |
| *SMC1A* | NM_006306.3:c.3103C>T | p.Arg1035* | Pathogenic |
| *STXBP1* | NM_003165.3:c.170-?_246+?del | Deletion (Exon 4) | Pathogenic |
| *SYNGAP1* | NM_006772.2:c.3233_3236delTCAG | p.Val1078Alafs*51 | Pathogenic |
| *TBC1D24^* | NM_001199107.1:c.172delC | p.Leu58* | Pathogenic |
| *TBC1D24^* | NM_001199107.1:c.845C>G | p.Pro282Arg | Pathogenic |
| *TSC2* | NM_000548.3:c.4508A>C | p.Gln1503Pro | Pathogenic |
| *UBE3A* | NM_130838.1:c.-44-?_*1888+?del | Deletion (Entire sequence) | Pathogenic |

*^^^Did not result in a molecular diagnosis since heterozygous pathogenic/likely pathogenic variant in a recessive gene*
